# Substance Use and Access to Health Care and Addiction Treatment among Homeless and Vulnerably Housed Persons in Three Canadian Cities

**DOI:** 10.1371/journal.pone.0075133

**Published:** 2013-10-04

**Authors:** Anita Palepu, Anne Gadermann, Anita M. Hubley, Susan Farrell, Evie Gogosis, Tim Aubry, Stephen W. Hwang

**Affiliations:** 1 Centre for Health Evaluation and Outcome Sciences, Department of Medicine, University of British Columbia, Vancouver, BC, Canada; 2 Department of Education and Counseling Psychology and Special Education, University of British Columbia, Vancouver, BC, Canada; 3 Department of Psychiatry, Royal Ottawa Health Care Group, University of Ottawa, Ottawa, ON, Canada; 4 Centre for Research on Inner City Health, Keenan Research Centre in the Li Ka Shing Knowledge Institute, St. Michael’s Hospital, Toronto, ON, Canada; 5 Centre for Research on Educational and Community Services, School of Psychology, University of Ottawa, Ottawa, ON, Canada; 6 Department of Medicine, University of Toronto, Toronto, Canada; Federal University of Rio de Janeiro, Brazil

## Abstract

**Introduction:**

We examined the prevalence of substance use disorders among homeless and vulnerably housed persons in three Canadian cities and its association with unmet health care needs and access to addiction treatment using baseline data from the Health and Housing in Transition Study.

**Methods:**

In 2009, 1191 homeless and vulnerably housed persons were recruited in Vancouver, Toronto, and Ottawa, Canada. Interviewer administered questionnaires collected data on socio-demographics, housing history, chronic health conditions, mental health diagnoses, problematic drug use (DAST-10≥6), problematic alcohol use (AUDIT≥20), unmet physical and mental health care needs, addiction treatment in the past 12 months. Three multiple logistic regression models were fit to examine the independent association of substance use with unmet physical health care need, unmet mental health care need, and addiction treatment.

**Results:**

Substance use was highly prevalent, with over half (53%) screening positive for the DAST-10 and 38% screening positive for the AUDIT. Problematic drug use was 29%, problematic alcohol use was lower at 16% and 7% had both problematic drug and alcohol use. In multiple regression models for unmet need, we found that problematic drug use was independently associated with unmet physical (adjusted odds ratio [AOR] 1.95; 95% confidence interval [CI] 1.43–2.64) and unmet mental (AOR 3.06; 95% CI 2.17–4.30) health care needs. Problematic alcohol use was not associated with unmet health care needs. Among those with problematic substance use, problematic drug use was associated with a greater likelihood of accessing addiction treatment compared to those with problematic alcohol use alone (AOR 2.32; 95% CI 1.18–4.54).

**Conclusions:**

Problematic drug use among homeless and vulnerably housed individuals was associated with having unmet health care needs and accessing addiction treatment. Strategies to provide comprehensive health services including addiction treatment should be developed and integrated within community supported models of care.

## Introduction

Homeless and vulnerably housed persons suffer from a high prevalence of chronic physical and mental illness, substance abuse, and mortality [1], [2], [3], [4], [5]. In relation to physical and mental health care they experience numerous barriers in accessing necessary health care and adhering to medications, and have lower life expectancy [6], [7], [8]. Drug and alcohol use are also associated with significant morbidity and mortality, as well as substantial service use and costs to various public service sectors including health, criminal justice, and social welfare [9], [10], [11], [12], [13].

Substance use has been recognized as a significant barrier to exiting homelessness and further exacerbates social marginalization [14], [15], [16], [17], [18]. Substance use among persons who are homeless has also been associated with early mortality [19], chronic physical illness, and longer periods of homelessness [20]. In addition, a substantial proportion of homeless individuals with substance use disorders also suffer from other mental disorders [21]. In a study of 1,191 homeless individuals in Toronto, Canada, 40% reported drug problems in the last 30 days and this was associated with significantly poorer mental health status [22]. Despite the extensive need for treatment of individuals with such dual or multiple diagnoses, many report unmet treatment needs and experience barriers to care [23].

The types of substances people use may affect their ability to access health care and addiction treatment, particularly for those who already face significant barriers to care because of their housing circumstances [24], [25]. The characterization of substance use and its relationship to accessing health care among the homeless and vulnerably housed in Canada is a critical first step to designing comprehensive interventions to improve their housing and health status. To date, research on the prevalence of substance use disorders in Canada among people experiencing homelessness or unstable housing has been limited to single cities [26], [27]. The current study extends the knowledge in this area by examining the prevalence of substance use disorders (including specific drug use patterns) among homeless and vulnerably housed persons in three large Canadian cities (Vancouver, Ottawa and Toronto) and its association with unmet health care needs and access to addiction treatment using baseline data from the Health and Housing in Transition Study.

## Materials and Methods

### Study Setting

We recruited participants from the Vancouver census metropolitan area (CMA), BC (population 2.33 million); Ottawa CMA, ON (population 1.22 million); and Toronto CMA, ON (population 5.62 million) (Statistics Canada 2010). On any given night, there are 2600 homeless individuals in Vancouver, over 1000 in Ottawa and as many as 5000 in Toronto [28], [29], [30].

### Target Population Recruitment

The sampling strategy and recruitment of the Health and Housing in Transition Study have been previously described in detail [31]. In brief, we recruited 1191 adult homeless and vulnerably housed participants based on a goal of 200 homeless and 200 vulnerably-housed participants from each of the three cities throughout 2009. A homeless participant was defined as living in a shelter, public space, motor vehicle, abandoned building, or not having their own place for which they paid rent. A vulnerably housed participant was defined as a person living in their own room or apartment or place, but had been homeless or had two or more moves over the past 12 months. The two stage sampling method used for recruiting homeless adults was adapted from Ardilly and Le Blanc [32].

Recruitment of homeless participants was done in shelters and meal programs. Participants who did not use shelters were recruited in meal programs proportionally to the number of homeless estimated to sleep on the street in each city. Shelters were randomly selected proportionally to the number of shelter beds at each site while meal programs were randomly selected based on their location and the number of meals served. At each shelter and meal program, the number of homeless individuals recruited ranged from 12 to 35 based on the number of recruitment sites in each city.

The stock of low-cost housing includes licensed and unlicensed rooming houses in Ottawa and Toronto and single room occupancy (SRO) hotels in Vancouver. Vulnerably housed participants were randomly selected from these housing settings in the different cities according to capacity. In addition to all licensed SROs and rooming houses, unlicensed sites were identified using multiple sources. Each city aimed to recruit 20 vulnerably housed individuals from 10 SROs (Vancouver) and 10 rooming houses each in Toronto and Ottawa to obtain a sample of 200 participants in each city. Due to challenges in gaining access to residents at these sites, our sampling plan for vulnerably housed participants was modified to include recruitment at meal programs, community health centers, and drop-in centers. Agency staff initially approached potential participants who were vulnerably housed to see if they were interested and trained research interviewers then screened the potential participants. The research interviewers administered the survey on-site in a private room in the shelter, SRO, rooming house, neighbourhood coffee shop or research office. Our final sample was comprised of 595 homeless and 596 vulnerably housed adults.

### Ethics Statement

Trained research interviewers explained the purpose of the study to eligible individuals and asked if they were interested in participating in the study. The research interviewer answered any questions that the potential participant had and if they agreed to participate, they were given two copies of the consent form to read and sign. Individuals were provided time to review the information in the consent form and ask any questions before being asked to give consent. To ensure that consent was informed for individuals with limited literacy, the research interviewer verbally reviewed the entire consent form. The participant retained one copy and the research interviewer retained the other copy. The participants were paid an honorarium ($20 CDN) following the interview. We obtained ethics approval from the Research Ethics Board at St. Michael’s Hospital (Toronto), the University of Ottawa, and the University of British Columbia (Vancouver). All potential participants who declined to participate or otherwise did not participate were eligible for any applicable treatment and services and were not disadvantaged in any other way by not participating in the study.

### Survey Instrument and Measures

Structured interviews lasted 60 to 90 minutes and contained validated measures as well as open-ended qualitative questions allowing participants to comment in more detail (See Hwang et al. for a full analysis and description of the survey instruments [31]). The survey included items and measures of socio-demographic characteristics, housing history over the past two years, chronic health conditions, lifetime prevalence of mental health diagnoses, access to and use of various health care services in the past 12 months, drug and alcohol use in the past 12 months, and incarceration history in the past 12 months. Drug use was defined as responding yes to the question: “In the past 12 months, have you used drugs other than those required for medical reasons?” Participants who responded affirmatively to this question were further asked which drugs they used in the past 12 months and the frequency of their use in the past month. We also used the 10-item Drug Abuse Screening Test (DAST-10) [33], [34] to screen for illicit drug use (positive screen≥3) and defined problematic drug use as a DAST-10 score ≥6, which would merit intensive assessment [35]. The Alcohol Use Disorders Identification Test (AUDIT) [36], a 10-item questionnaire designed to screen for alcohol use disorder (positive screen≥8) and we defined problematic alcohol use as an AUDIT score of ≥20 [37]. These instruments have been validated for use in previous studies of vulnerable populations [35], [38], [39], [40]. We defined unmet physical health care needs as responding yes to the question: “During the past 12 months, was there ever a time when you felt that you needed health care but you didn’t receive it?” [41]. Unmet mental health care need was defined as responding affirmatively to the question: “Have you needed mental health care in the past 12 months but were not able to get help?” These questions were based on the definition of unmet health care needs used in other population-based, national surveys (e.g., Canadian Community Health Survey-CCHS, National Health Interview Survey, Joint Canada/US Survey of Health) [42], [43]. There were two questions addressing addiction treatment: “In the past 12 months, have you followed a program, been in therapy, or taken part in self-help groups for alcohol use problems?” and “In the past 12 months, have you followed a program, been in therapy, or taken part in self-help groups for drug use problems?” The responses to these two questions comprised our dichotomous participation in addiction treatment variable such that participation in either or both types of treatment was recorded as “Yes” to engaging in addiction treatment in the past 12 months.

### Statistical Analysis

We combined the data for the homeless and vulnerably housed persons because previous analysis has shown that they are comparable in many baseline characteristics, which is a function of our recruitment strategy [31]. Comparisons were made using the chi-square test or Fisher exact test (where appropriate) for categorical variables and one-way ANOVA for continuous variables. For missing data (i.e., participant did not know or refused to answer), the denominators were adjusted accordingly. We conducted three multiple logistic regressions to examine the independent association of problematic drug use and problematic alcohol use with the following service use variables: unmet physical health care need, unmet mental health care need, and addiction treatment. For the outcome variable addiction treatment, only participants with scores on the DAST-10≥6 and/or AUDIT≥20 were included in the analysis. To compare respondents with problematic drug use, problematic alcohol use, and both problematic drug and alcohol use with regard to accessing addiction treatment, the following dummy variable with three categories was created and entered in the regression: Only problematic drug use (DAST-10≥6, but not AUDIT≥20); only problematic alcohol use (AUDIT≥20, but not DAST-10≥6), and both problematic drug and alcohol use (AUDIT≥20 and DAST-10≥6). We adjusted for housing status at recruitment, lifetime duration of homelessness, gender, age, self-identified racial/cultural group, employment, incarceration, city of recruitment, number of chronic health conditions, ever diagnosed with a mental health problem, and having a primary care provider. For each of the logistic regression analyses, we included participants who had complete data on all variables of interest (i.e., listwise deletion was used). We also conducted sub-analyses for each city and fit three multiple logistic regression models for the outcomes above.

## Results

At the end of our baseline data collection, we recruited 1191 homeless or vulnerably housed participants of whom 396 (33.3%) were from Vancouver, 399 (33.4%) were from Toronto and 396 (33.3%) were from Ottawa. Table 1 provides characteristics of the sample stratified by city. The majority of respondents were single/never married (58%), white (63%), and had completed some high school (45%). Significant differences across cities were found for several demographic characteristics, such as age, gender, marital status, self-identified racial/cultural group, employment in the past 12 months, monthly income, and lifetime duration of homelessness. Significant differences across cities were also noted with regard to health status. Respondents in Vancouver reported a higher number of chronic health conditions compared to Ottawa and Toronto. A higher proportion of respondents in Ottawa reported having ever been diagnosed with a mental health problem compared to Vancouver and Toronto (60% vs. 54% and 41%, p<0.01).

**Table 1:** Characteristics of homeless and vulnerably housed participants across Vancouver, Toronto, and Ottawa.

Variable	Vancouver (n = 396)	Toronto (n = 399)	Ottawa (n = 396)	p-value
Age (years), mean (SD)	42.0 (10.2)	43.5 (9.9)	41.1 (11.4)	<0.01
Gender, n (%)				0.01
Male	244 (62)	258 (65)	278 (70)	
Female	140 (36)	132 (33)	117 (30)	
Transgender	9 (2)	9 (2)	0	
Housing Status, n (%)				0.98
Vulnerably housed	199 (50)	198 (50)	199 (50)	
Homeless	197 (50)	201 (50)	197 (50)	
Marital status, n (%)				0.02
Single/never married	203 (52)	247 (62)	236 (60)	
Divorced/separated	110 (28)	101 (26)	98 (25)	
Widowed	15 (4)	9 (2)	6 (1)	
Married/common-law	36 (9)	24 (6)	22 (6)	
Partnered, not married	27 (7)	17 (4)	33 (8)	
Racial/cultural group, n (%)				<0.01
White	222 (58)	203 (54)	297 (76)	
Black/African-Canad.	12 (3)	68 (18)	26 (7)	
First Nations/Aborig.	105 (27)	52 (14)	47 (12)	
Mixed ethnicity	30 (8)	24 (6)	10 (2)	
Other	14 (4)	32 (8)	13 (3)	
Highest level of education, n (%)				0.51
Some high school	178 (46)	170 (43)	181 (46)	
Completed high school or equivalent	99 (25)	90 (23)	87 (22)	
Some post-secondary education or higher	114 (29)	137 (34)	126 (32)	
Employed in past 12 months, n (%)	170 (43)	137 (34)	166 (42)	0.02
Monthly income, (CDN dollars) median (Q1–Q3)	1074 (682–1787)	770 (500–1252)	825 (512–1307)	0.02
Lifetime duration of homelessness (years), median(Q1–Q3)	3.2 (1.1–6.7)	3.0 (1.1–8.0)	2.1 (1.0–5.8)	0.04
Incarcerated in past 12 months, n (%)	109 (28)	105 (26)	123 (31)	0.29
Number of chronic health conditions^1^, n (%)				<0.01
0	29 (7)	80 (20)	42 (10)	
1	80 (20)	84 (21)	86 (22)	
2	63 (16)	75 (19)	59 (15)	
≥3	224 (57)	160 (40)	209 (53)	
Ever diagnosed with a mental health problem, n (%)	210 (54)	161 (41)	235 (60)	<0.01

1 Chronic health conditions include the following conditions that the participant has had for 6 months or more that have been diagnosed by a health professional: high blood pressure; heart disease; asthma; COPD (includes emphysema and chronic bronchitis); cirrhosis; Hepatitis B or C; intestinal or stomach ulcers; urinary incontinence; bowel disorders; arthritis; problems walking, lost limb, or other physical handicap; HIV/AIDS; epilepsy; fetal alcohol syndrome or fetal alcohol spectrum disorder; head injury; glaucoma; cataracts; cancer, diabetes; or anemia.

As presented in Table 2, Vancouver participants reported the highest prevalence of any drug use in the previous 12 months compared to Toronto and Ottawa (82% vs. 59% and 74%, p<0.01). The distribution of types of drugs used differed by city, with a significantly higher prevalence of use in Vancouver for amphetamines/crystal meth, cocaine/crack, heroin, and heroin combined with cocaine (speedballs). Prescription narcotic use was higher in Ottawa relative to the other cities. A higher proportion of the sample screened positive for problematic drug use in Vancouver compared to Toronto and Ottawa (37% vs. 22% and 28%, p<0.01). There was no difference in the proportion of participants who screened positive for problematic alcohol use based on the AUDIT by city. Overall, the prevalence of problematic drug use (DAST-10≥6) was 29%; problematic alcohol use (AUDIT≥20) was lower at 16%, and 7% had both problematic drug and alcohol use.

**Table 2:** Substance use in the past 12 months by city.

Variable	Vancouver n = 396 n (%)	Toronto n = 399 n (%)	Ottawa n = 396 n (%)	p-value
Drug use in past 12 months^1^	321 (82)	233 (59)	291 (74)	<0.01
Amphetamines/crystal methamphetamine^1^	89 (23)	30 (8)	33 (8)	<0.01
Benzodiazepines^1^	48 (12)	40 (10)	38 (10)	0.46
Cocaine/crack^1^	252 (64)	162 (41)	185 (47)	<0.01
Heroin alone^1^	103 (26)	28 (7)	31 (8)	<0.01
Combined heroin and cocaine (speedballs)^1^	55 (14)	15 (4)	8 (2)	<0.01
Prescription narcotics^1^	77 (20)	62 (16)	90 (23)	0.03
DAST-10 screen positive^2^	260 (66)	163 (41)	209 (53)	<0.01
Problematic drug use (DAST-10≥6)	144 (37)	87 (22)	110 (28)	<0.01
Alcohol ≥4 times/week^3^	56 (14)	68 (17)	65 (17)	0.50
AUDIT screen positive^4^	144 (37)	149 (37)	155 (39)	0.69
Problematic alcohol use (AUDIT≥20)	60 (15)	59 (15)	68 (17)	0.59

1 Any use of drugs in the past 12 months for non-medical reasons.

2 DAST-10: Drug Abuse Screening Test (positive screen≥3).

3 Use of alcohol ≥4 times/week in the past 12 months.

4 AUDIT: Alcohol Use Disorders Identification Test (positive screen≥8).

Note. Due to missing data there were slightly varying denominators.

In the two multiple logistic regression models that focused on unmet needs (Tables 3–4), we found that problematic drug use was independently associated with unmet physical (adjusted odds ratio [AOR] 1.95; 95% confidence interval [CI] 1.07–1.88) and unmet mental (AOR 3.06; 95% CI 2.17–4.30) health care needs, controlling for city, demographic, health, and health care characteristics. Problematic alcohol use was not associated with unmet physical or mental health care needs. Other factors that were independently associated with unmet physical or mental health care needs included having ≥3 self-reported chronic health conditions, having ever been diagnosed with a mental health problem, and being homeless at recruitment. Furthermore, not having a primary care provider was independently associated with unmet physical health care needs.

**Table 3:** Multiple logistic regression model of independent associations between problematic substance use and unmet physical health care needs.

	Have unmet needs forphysical health care inpast 12 months (n = 445)	No unmet needs forphysical health care inpast 12 months (n = 736)	Adjusted OR for having unmet needs for physical health care in past 12 months (95% CI) (n = 1111)
Problematic drug use^1^, n (%)	170 (38)	165 (23)	**1.95 (1.43, 2.64)**
Problematic alcohol use^2^, n (%)	77 (17)	106 (15)	1.02 (0.71, 1.46)
Housing status, n (%)			
Vulnerably housed (ref)	225 (51)	364 (49)	1.00
Homeless	220 (49)	372 (51)	1.03 (0.79, 1.34)
Lifetime duration of homelessness (in years), mean (SD)	5.5 (6.3)	4.9 (5.8)	1.00 (0.98, 1.02)
Gender, n (%)			
Female (ref)	154 (35)	232 (32)	1.00
Male	286 (64)	487 (66)	1.01 (0.75, 1.37)
Transgender	4 (1)	14 (2)	0.55 (0.17, 1.82)
Age (in years), mean (SD)	41.6 (10.5)	42.6 (10.6)	1.00 (0.98, 1.01)
Racial/cultural group, n (%)			
White (ref)	282 (65)	430 (61)	1.00
Black/African-Canadian	33 (8)	73 (10)	0.89 (0.55, 1.46)
First Nations/Aboriginal	79 (18)	125 (17)	0.93 (0.65, 1.34)
Mixed ethnicity	30 (7)	34 (5)	1.53 (0.87, 2.68)
Other	10 (2)	49 (7)	0.51 (0.24, 1.07)
Employed in past 12 months, n (%)	186 (42)	282 (38)	1.23 (0.93, 1.62)
Incarcerated in past 12 months, n (%)	151 (34)	183 (25%)	1.11 (0.83, 1.50)
City, n (%)			
Vancouver (ref)	160 (36)	231 (32)	1.00
Toronto	136 (31)	260 (35)	1.02 (0.73, 1.42)
Ottawa	149 (33)	245 (33)	0.87 (0.63, 1.20)
Number of chronic health conditions^3^, n (%)			
0 (ref)	30 (7)	120 (16)	1.00
1	69 (15)	179 (24)	1.36 (0.81, 2.30)
2	65 (15)	130 (18)	1.69 (0.98, 2.91)
≥3	281 (63)	307 (42)	3.58 (2.20, 5.83)
Ever diagnosed with a mental health problem, n (%)	260 (59)	340 (47)	1.39 (1.06, 1.82)
Has a primary care provider, n (%)	252 (57)	461 (63)	0.56 (0.43, 0.75)

Note: Coefficients are based on a multiple logistic regression model. Bolded coefficients are significant at the p<0.05 level. Nagelkerke R^2^ = 0.14.

Due to missing data, there were slightly varying denominators for reported frequencies.

OR: Odds ratio CI: Confidence interval.

1 DAST-10≥6.

2 AUDIT≥20.

3 For a definition of chronic health conditions please see footnote of table 1.

**Table 4:** Multiple logistic regression model of independent associations between problematic substance use and unmet mental health care needs.

	Have unmet needs formental health care inpast 12 months (n = 278)	No unmet needs formental health care inpast 12 months (n = 906)	Adjusted OR for having unmetneeds for mental health care inpast 12 months (95% CI) (n = 1115)
Problematic drug use^1^, n (%)	143 (52)	198 (22)	**3.06 (2.17, 4.30)**
Problematic alcohol use^2^, n (%)	57 (21)	129 (14)	1.27 (0.85, 1.91)
Housing status, n (%)			
Vulnerably housed (ref)	135 (49)	456 (50)	1.00
Homeless	143 (51)	450 (50)	**1.45 (1.06, 1.98)**
Lifetime duration of homelessness (in years), mean (SD)	5.4 (6.2)	5.0 (6.0)	0.99 (0.96, 1.02)
Gender, n (%)			
Female (ref)	103 (37)	285 (32)	1.00
Male	167 (61)	607 (67)	1.11 (0.78, 1.58)
Transgender	6 (2)	12 (1)	1.39 (0.42, 4.56)
Age (in years), mean (SD)	40.9 (9.7)	42.6 (10.8)	0.99 (0.98, 1.01)
Racial/cultural group, n (%)			
White (ref)	180 (67)	538 (61)	1.00
Black/African-Canadian	13 (5)	93 (10)	0.73 (0.37, 1.45)
First Nations/Aboriginal	52 (19)	150 (17)	0.96 (0.62, 1.47)
Mixed ethnicity	12 (5)	51 (6)	0.69 (0.33, 1.43)
Other	11 (4)	48 (6)	1.15 (0.52, 2.55)
Employed in past 12 months, n (%)	107 (39)	362 (40)	1.07 (0.77, 1.50)
Incarcerated in past 12 months, n (%)	102 (37)	235 (26)	1.23 (0.88, 1.73)
City, n (%)			
Vancouver (ref)	108 (39)	286 (32)	1.00
Toronto	66 (24)	330 (36)	0.71 (0.47, 1.06)
Ottawa	104 (37)	290 (32)	0.88 (0.61, 1.23)
Number of chronic health conditions^3^, n (%)			
0 (ref)	19 (7)	131 (15)	1.00
1	34 (12)	214 (24)	0.91 (0.46, 1.82)
2	42 (15)	154 (17)	1.64 (0.82, 3.28)
≥3	183 (66)	407 (45)	**2.67 (1.43, 4.98)**
Ever diagnosed with a mental health problem, n (%)	206 (75)	398 (45)	**2.98 (2.12, 4.20)**
Has a primary care provider, n (%)	175 (63)	541 (60)	0.80 (0.57, 1.12)

Note: Coefficients are based on a multiple logistic regression model. Bolded coefficients are significant at the p<0.05 level. Nagelkerke R^2^ = 0.23.

Due to missing data, there were slightly varying denominators for reported frequencies.

OR: Odds ratio; CI: Confidence interval.

1 DAST-10≥6.

2 AUDIT≥20.

3 For a definition of chronic health conditions please see footnote of table 1.

In the third multiple logistic regression model (Table 5), which only included participants with problematic substance use, we found that compared to problematic alcohol use only, those with problematic drug use only (AOR 2.80; 95% CI 1.60–4.89) and those with problematic drug and alcohol use (AOR 2.32; 95% CI 1.18–4.54) were independently associated with accessing addiction treatment. Being homeless at recruitment (1.67; 95% CI: 1.09–2.56), and having a primary care provider (AOR 1.86; 95% CI 1.18–2.94) were also positively associated with addiction treatment. The analyses stratified by city are generally consistent with the overall sample but with some associations losing their statistical significance due to lower sample sizes (detailed results available from authors on request).

**Table 5:** Multiple logistic regression model of associations between type of substance use with addiction treatment among persons with problematic substance use.

	No addiction treatment in past 12 months (n = 193)	Use of addiction treatment in past 12 months (n = 250)	Adjusted OR for having been in addiction treatment in past 12 months (95% CI) (n = 421)
Type of substance use, n (%)			
Problematic alcohol use only^1^ (ref)	66 (44.0)	37 (14.8)	1.00
Problematic drug use only^2^	91 (47.2)	165 (66.0)	**2.80 (1.60, 4.89)**
Problematic alcohol and drug use^3^	36 (18.7)	48 (19.2)	**2.32 (1.18, 4.54)**
Housing status, n (%)			
Vulnerably housed (ref)	119 (62)	128 (51)	1.00
Homeless	74 (38)	122 (49)	**1.67 (1.09, 2.56)**
Lifetime duration of homelessness (in years), mean (SD)	6.1 (6.8)	5.6 (6.2)	0.99 (0.95, 1.02)
Gender, n (%)			
Female (ref)	68 (35)	89 (36)	1.00
Male	123 (64)	155 (62)	1.24 (0.76, 2.05)
Transgender	2 (1)	6 (2)	2.20 (0.38, 12.87)
Mean Age (SD)	39.7 (9.8)	39.7 (9.4)	1.01 (0.99, 1.04)
Racial/cultural group, n (%)			
White (ref)	110 (59)	159 (65)	1.00
Black/African-Canadian	7 (4)	13 (5)	1.22 (0.43, 3.41)
First Nations/Aboriginal	57 (30)	46 (19)	0.58 (0.33, 1.00)
Mixed ethnicity	6 (3)	19 (8)	1.88 (0.68, 5.25)
Other	7 (4)	7 (3)	0.62 (0.19, 2.00)
Employed in past 12 months, n (%)	74 (39)	95 (38)	0.90 (0.56, 1.43)
Incarcerated in past 12 months, n (%)	80 (42)	109 (44)	1.08 (0.69, 1.67)
City, n (%)			
Vancouver (ref)	70 (36)	105 (42)	1.00
Toronto	53 (28)	66 (26)	0.82 (0.48, 1.43)
Ottawa	70 (36)	79 (32)	0.76 (0.45, 1.30)
Number of chronic health conditions^4^, n (%)			
0 (ref)	19 (10)	11 (4)	1.00
1	30 (15)	56 (23)	2.37 (0.92, 6.09)
2	32 (17)	42 (17)	1.77 (0.68, 4.62)
≥3	11239 (583)	141 (56)	1.86 (0.77, 4.47)
Ever diagnosed with a mental health problem, n (%)	104 (55)	160 (65)	1.40 (0.89, 2.20)
Has a primary care provider, n (%)	112 (58)	181 (72)	**1.86 (1.18, 2.94)**

Note: Coefficients are based on a multiple logistic regression model. Bolded coefficients are significant at the p<0.05 level. Nagelkerke R^2^ = 0.16.

Due to missing data, there were slightly varying denominators for reported frequencies.

OR: Odds ratio; CI: Confidence interval.

1 AUDIT≥20, but not DAST-10≥6.

2 DAST-10≥6, but not AUDIT≥20.

3 AUDIT≥20 and DAST-10≥6.

4 For a definition of chronic health conditions please see footnote of table 1.

## Discussion

Problematic drug use among homeless and vulnerably housed persons in our sample was associated with unmet physical and mental health care needs. Having a higher burden of chronic health conditions as well as ever being diagnosed with a mental health problem were also independently associated with unmet health care needs. Given the premature mortality among homeless and vulnerably housed persons [8], it is of concern that having a higher comorbidity burden was associated with unmet health care needs. Other studies have also found that having lower physical and mental health scores on the SF-12 [44] and having two or more chronic medical comorbidities was associated with unmet health care needs [45]. In contrast, one study found that, among homeless persons, having a chronic medical condition was positively associated with having a family doctor as their usual source of care although the odds significantly decreased with each additional year spent homeless in the respondent’s lifetime [25]. Further studies are needed to examine whether those who are not able to receive care are more likely to accumulate a higher burden of chronic health conditions. Concurrent mental illness and substance use is highly prevalent among homeless populations and this subgroup is often the most marginalized [21], [23]. Engagement in primary care reduced the likelihood of reporting unmet physical health care need but was not associated with a decreased likelihood of unmet mental health care need. The development and evaluation of efficacious models for delivering concurrent treatment services (for addiction and mental illness) that are accessible to the homeless population are urgently needed [46].

Among those with a problematic substance use disorder, problematic drug use (DAST-10≥6) was associated with a greater likelihood of accessing addiction treatment compared to those with problematic alcohol use (AUDIT≥20). Furthermore, having a primary care provider was positively associated with accessing addiction treatment and highlights the importance of engagement in care. This improved access to addiction treatment is critical given that substance use among persons who are homeless and vulnerably housed can be a barrier to residential stability and engagement in mental and physical health care [16], [20], [47], [48]. Interestingly, being homeless was associated with accessing addiction treatment. It may be that people at shelters are more likely to have contact with case managers and counselors who try to connect them to addiction treatment compared to persons who are vulnerably housed.

Our study had some limitations. Given that we do not know the characteristics or true size of the population of persons who are homeless and vulnerably housed in these three cities (or in Canada), it was not possible to construct a comprehensive sampling frame, and therefore our sample may not be representative. However, we did use sampling strategies comparable to those described in other studies of this population. Although we used the DAST-10 and the AUDIT, which are validated screening tools for problematic drug and alcohol use, including in vulnerable populations, there may have been social desirability bias in the reporting of drug and alcohol use resulting in an underestimation of prevalence levels. In addition, individuals who were under the influence of alcohol or drugs at the time of recruitment were excluded because they were unable to provide informed consent. The presence of chronic health conditions and mental health disorders was determined by self-report that may also lead to an under estimation of prevalence rates. Our measure of unmet physical health care need did not explicitly specify physical health and it is possible that some participants may have included mental health needs when responding to this question. However, this question was derived from the definition of unmet health care needs used in other population-based, national surveys [42], [43]. Finally, due to the cross-sectional nature of the data, we are unable to infer causation, rates of relapse, or use of follow-up care. Despite these limitations, this is the only study to date to have included such a large sample of homeless and vulnerably housed persons and to recruit from three cities of varying sizes in Canada.

## Conclusions

In summary, we found that, among homeless and vulnerably housed individuals, problematic drug use was associated with accessing addiction treatment and having unmet health care needs. The fact that homeless and vulnerably housed individuals with problematic drug use recognize that they have unmet needs for care suggests that they will engage with services if they are provided in an appropriate context. Given the complex health and social situation of this vulnerable group, strategies to provide coordinated, comprehensive health care services that include addiction treatment should be developed and integrated within supportive and supported housing and other community support models of care. Outreach services may provide a variety of access points for these individuals with the goal of engaging this population into care. Based on the findings of this study, policymakers and planners should develop integrated services that are easily accessible and appropriate for homeless and vulnerably housed individuals to close the gap between need and treatment. Such programs would help address their physical, mental and addiction disorders while at the same time assist them to re-establish residential stability, which may decrease the possibility of relapse and improve access to other important resources [49].
